# Mechanisms of systemic resistance to pathogen infection in plants and their potential application in forestry

**DOI:** 10.1186/s12870-023-04391-9

**Published:** 2023-08-25

**Authors:** S. K. Wilson, T. Pretorius, S. Naidoo

**Affiliations:** Department of Biochemistry, Genetics and Microbiology, Forestry and Agricultural Biotechnology Institute, University of Pretoria, Pretoria, 0028 South Africa

**Keywords:** Plant-pathogen interactions, Phytohormones, Systemic resistance, Priming

## Abstract

**Background:**

The complex systemic responses of tree species to fight pathogen infection necessitate attention due to the potential for yield protection in forestry.

**Results:**

In this paper, both the localized and systemic responses of model plants, such as *Arabidopsis* and tobacco, are reviewed. These responses were compared to information available that investigates similar responses in woody plant species and their key differences were highlighted. In addition, tree-specific responses that have been documented were summarised, with the critical responses still relying on certain systemic acquired resistance pathways. Importantly, coniferous species have been shown to utilise phenolic compounds in their immune responses. Here we also highlight the lack of focus on systemic induced susceptibility in trees, which can be important to forest health.

**Conclusions:**

This review highlights the possible mechanisms of systemic response to infection in woody plant species, their potential applications, and where research may be best focused in future.

## Background

Woody plants are critical to pulp and paper production worldwide but are threatened by feeding and infection by various pests and pathogens. Upon the induction of biotic threat, plants elicit a localized response, through phytohormone signalling, to combat attack at the site of infection [1]. A systemic response in distal tissues may also be stimulated through an integrated web of systemic acquired resistance and induced systemic resistance mechanisms [1, 2]. These basal defences serve as the frontline for the plant’s protection against future biotic challenges.

Even in plants that share the same basal defence pathways, a susceptible host may activate these defences too late, or at too low a level upon pest or pathogen attack [3]. In addition to preformed barriers, plants may activate induced resistance (IR) in response to a specific pathogen. The ability of a plant to alter its defence repertoire may lead to defence against current and future infections [4, 5]. The immune responses during these interactions, however, are highly complex and often differ between plants as they rely on the pathogen’s lifestyle and mode of infection [6]. These efforts made to fight infection can have long-lasting effects on plant immunity by priming responses against future infection or attack.

This phenomenon of systemic plant resistance was pioneered by the work of Joseph Kuć. He identified the involvement of oxalate [7], chitinases and peroxidases [8], among other metabolites and proteins, in the induction of systemic defences in plants. Many studies have since built upon this foundation, which have further reinforced the mechanisms of induced systemic responses in plants, as reviewed by Vlot et al. [9]. However, many questions remain, such as how and when systemic signalling is activated and how independent the different classes of systemic responses are. The lifestyle of tree species is fundamentally different to model organisms and trees often harbour unique secondary metabolites for host defence. This review explores the current knowledge about responses of plants to infection in systemic tissues, first focusing on mechanisms established in model organisms, and secondly on the less well-documented woody plants systemic responses to infection. Gaps are highlighted in the understanding of how woody plants tailor their systemic signalling mechanisms to magnify defences against future biotic stress challenges.

## Localized responses in model organisms

To mount an effective defence against any pathogen, a plant must elicit the correct signalling pathways early in infection. Depending on the lifestyle of the pathogen, infection induces the plant to produce single or multiple hormone signals [10]. Evidence in model plants indicate that the salicylic acid (SA), jasmonic acid (JA) and ethylene (Et) pathways are strongly linked to the specificity of plant defence, as revealed by studies in their biosynthesis and downstream signaling during infection [6, 11]. SA provides plants with protection against biotrophic pathogens, which grow in and obtain nutrients from living cells, while JA and Et work together against necrotrophic organisms [6, 12], which grow in and obtain nutrients from dead or dying cells [6].

Upon recognition of an invading pathogen, host cells surrounding the site of infection receive communication of which defences should be activated. Phytohormone signalling consists of molecules that are rapidly induced before, during and after recognition. At the same time, non-specific signals, including reactive oxygen species (ROS), mitogen-activated protein kinases (MAPKs) and calcium ions (Ca^2+^), further aid in the induction of plant defence [13]. These responses are well-known in model species and have been reviewed in depth, thus this review will only provide a brief overview.

### The salicylic acid pathway

For many years, SA has been known to induce resistance against plant diseases [14]. In *Arabidopsis*, the *non-expressor of PR1* (*NPR1*) gene plays an important role in the activation of SA-responsive gene expression. The BTB/POZ domain and transcriptional activation motifs within NPR1 allow for its pivotal role in defence through the binding of TGA transcription factors (TFs) [15]. TGA2/5/6 factors are responsible for activating many downstream defence responses including the hypersensitive response (HR), pattern-triggered immunity (PTI), effector-triggered immunity (ETI) and, importantly, systemic responses [16]. Various WRKY TFs are also dependent on NPR1 in their positive and negative regulation of SA-responsive genes [17].

Upon pathogen challenge, the rapid increase in SA induces an oxidative burst that dissociates NPR1 into monomers [18]; it undergoes a conformational change that allows it to bind to SA which promotes NPR1 monomerization and transport to the nucleus [15]. The release of the NPR1 BTB/POZ inhibitory domain following the conformational change facilitates downstream gene activation [19]. The most common response downstream of NPR1 is the accumulation of pathogenesis-related proteins (PRs), which are collectively induced upon infection. First reported in *Nicotiana tabacum* [20], these proteins form various classes of antimicrobials [21]. PR1 (cysteine-rich secretory protein-related), PR2 (β-1,3-glucanase) and PR5 (thaumatin-like protein), along with their encoded domains, are used as reliable markers for a SA-associated response [22]. Another typical response of SA induction is HR, aided by ROS, which results in localized cell death at the site of infection to restrict a pathogens movement [23].

### The jasmonic acid and ethylene pathways

Unlike SA, JA does not act as the inducer of downstream effects. Downstream plant responses rely on the interaction between the F-Box protein, CORONATINE INSENSITIVE 1 (COI1), and JASMONATE ZIM-domain containing transcriptional repressors (JAZ), which act as the receptor for JA-Ile, the bioactive form of JA [24, 25]. Downstream, the degradation of JAZ proteins triggers the release of the TF MYC2, which transcriptionally activates genes responsive to JA [26–28]. MYC2 has also been shown to influence SA-mediated defences, as it has dual regulatory roles in several points in the SA pathway [29]. This highlights the complex nature of crosstalk in plant immunity, as discussed below.

Mutually antagonistic to one other, MYC2 and Ethylene-Response-Factor 1 (ERF1) are induced upon wounding and necrotrophic pathogen infection, respectively. During wounding or herbivore attack, MYC2 activates genes, such as *vegetative storage protein* (*VSP*) [30]. Upstream, several lipoxygenase genes, such as *LOX3*, contribute to JA production in response to wounding [31, 32]. In contrast, the response to necrotrophic pathogens results in the induction of several AP2/ERF domain TFs. For example, TFs ERF1 and ORA59 work together with MED25 [33] to mediate this response through the production of proteins like plant defensin 1.2 (PDF1.2) [34, 35].

### Crosstalk between salicylic acid, jasmonic acid/ethylene and other phytohormones

While the role of SA and JA/Et pathways in plant defence are well known, other major hormones including abscisic acid (ABA), gibberellins, auxins, cytokinins, brassinosteroids and strigolactones also play a role in plant defence [36–38]. Hormonal crosstalk is particularly important when it comes to multi-attacker situations, as it allows the plant to prioritize the correct defence pathways while limiting the cost of fitness associated with induced immunity [39].

It is generally viewed that SA and JA/Et act antagonistically to one another to provide resistance to biotrophic and necrotrophic pathogens respectively [6]. While most studies have shown these pathways to be antagonistic, neutral and synergistic interactions do exist, as reviewed by Aerts et al. [40]. One of these studies showed that *Arabidopsis* leaves, treated with SA, MeJA, or both, resulted in co-clustering of hormone profiles across all three conditions, providing evidence for the coregulation of SA and JA suggestive of their synergistic interaction [41]. Both positive and negative regulators at multiple steps in either pathway serve as potential targets for the crosstalk, and these have been thoroughly reviewed [42, 43].

## Systemic responses in model organisms

Not only does IR activate defence at the localized tissues, but it also activates systemic responses in distal undamaged tissues, termed systemic acquired resistance (SAR) [44]. This serves as a form of longer-term immunity in distal tissues to prevent future infection by the same pathogen. The resulting immunity is typically effective against biotrophic pathogens, lasts anywhere from days to an entire lifetime, and is transgenerational [45, 46]. Systemic responses elicited by root–associated beneficial bacteria and fungi is termed induced systemic resistance (ISR) and shares many features with the SAR pathway [47].

### Systemic acquired resistance

#### Localized events and mobile signalling in systemic acquired resistance

The establishment of SAR requires that signals generated at the site of infection are transported to and subsequently recognised in systemic tissues, typically via PTI and ETI [48]. The current model for SAR suggests that various compounds fall either under the regulation of SA or act independently in other pathways [49, 50]. In addition to the compounds discussed below that physically move through the vascular tissue, volatile compounds, such as methyl-SA (MeSA) and monoterpenes, also move to distal tissues indirectly through the air [51–53].

**Fig. 1:**
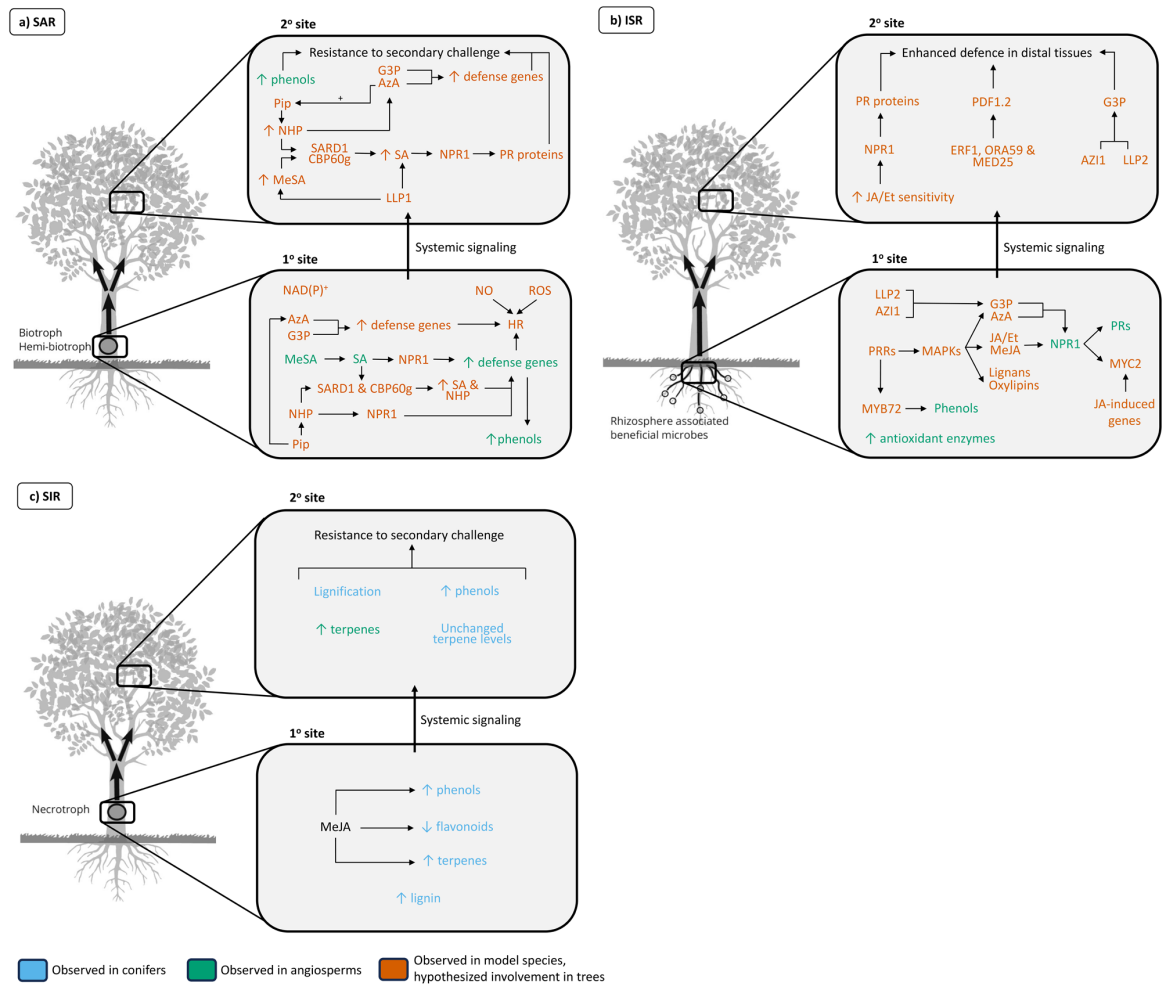
Observed metabolic and molecular responses from different tree and model species interactions, including **(a)** Systemic acquired resistance, **(b)** induced systemic resistance, and **(c)** systemic induced resistance. SAR: systemic acquired resistance; ISR: induced systemic resistance; SIR: systemic induced resistance; HR: hypersensitive response; MeSA: methyl-salicylic acid; Pip: pipecolic acid; NHP: N-hydroxypipecolic acid; AzA: azelaic acid; G3P: glycerol-3-phosphate; SA: salicylic acid; PR: pathogenesis-related proteins; JA: jasmonic acid; MeJA: methyl-jasmonate; JA/Et: jasmonic acid/ethylene; NPR1: NONEXPRESSOR OF PR GENES 1; NO: nitric oxide; ROS: reactive oxygen species; PR proteins: pathogenesis-related proteins; SARD1: SAR deficient 1; CBP60g: calmodulin-binding protein 60 g; MAPK: mitogen-activated kinase; LLP: legume lectin-like protein; PDF1.2: plant defensin 1.2; ORA59: octadecanoid-responsive AP2/ERF 59; MED25: MEDIATOR25; AZI1: azelaic acid induced 1. Positive feedback is indicated by +

While SA is transported through the vascular tissue as a possible mobile signal [54], it may also play a role in the perception and/or propagation of the mobile signal [55, 56]. Regardless, the induction of SAR relies heavily on either the synthesis or accumulation of SA, and sometimes both, in systemic tissues [57]. In the past, MeSA has been shown to be a notable propagator of SAR (Fig. 1a); its conversion to SA is useful to the induction of systemic responses to infection [9]. However, in *Arabidopsis* inoculated with *Pseudomonas syringae*, very little MeSA that was produced was retained in plant tissue [58] and in *Arabidopsis* lines wherein the production of MeSA was knocked out, SAR was not compromised [58]. Thus, it appears that MeSA is associated with SAR, but is not a driving force behind it.

SA is not the only phytohormone involved in the induction of SAR; Pipecolic acid (Pip) is rapidly induced and is linked to the systemic accumulation of SA necessary for SAR [59, 60]. Once induced by the increase in SA due to pathogen challenge, Pip is synthesized in two reactions mediated by AGD2-like Defence Response Protein 1 (ALD1) and SAR-DEFICIENT 4 (SARD4) [60–62]. Importantly, while the production of Pip is not required for signal generation, its production via ALD1 is necessary for perception in systemic tissues [63]. Pip serves as a secondary messenger for SA in SAR in order to promptly trigger other responses in distal tissues. In parallel with SA, Pip regulates ROS and nitric oxide (NO) to further activate SAR [63].

Pip is a biosynthetic precursor of N-hydroxypipecolic acid (NHP), another key player in SAR that serves as the bioactive signal in the Pip/NHP signaling pathway ([62], [43]; Fig. 1a). The induction of SAR by NHP occurs via the accumulation of both NHP and Pip, however Pip must be converted to bioactive NHP by flavin mono-oxygenase 1 (FMO1; 64). This consequently stimulates the activity of TGA TFs, which promote the action of SYSTEMIC ACQUIRED RESISTANCE DEFICIENT 1 (SARD1) and CALMODULIN-BINDING PROTEIN 60 g (CBP60g) [65]. Notably, NHP and SA work concurrently to facilitate a stronger activation of SAR [66, 67]. The SA receptor NPR1 is also critical to the NHP-triggered SAR and transcriptional responses. While Pip is a mobile signal for SAR, NHP functions as an inducer of defence responses. Zeier [68] has thoroughly reviewed the downstream effects of NHP in systemic resistance.

Previous research has suggested that the SA, NHP and azelaic acid (AzA) pathways run parallel to each other [69]. However, latest findings suggest that the SA and Pip pathways are interdependent and act synergistically [9]. SARD1 and CBP60g, under the control of CALMODULIN BINDING TF 1–3 (CAMTA), regulate the biosynthesis and positive feedback of both SA and NHP biosynthesis [70, 71]. There is evidence to support that the accumulation of NHP-induced NPR1 can be involved in regulating the synthesis of SA, NHP and their regulators, SARD1 and CBP60g [70]. Also reviewed by Zeier is the mechanism by which SAR is terminated via glycosylation of both NHP and SA to maintain a primed immune state [68]. Both phytohormones undergo glycosylation by the same glycosyltransferase to produce inactive glucosides of NHP and SA to terminate SAR. These glucosides keep plants in an uninduced, basal immune state in preparation for future pathogen attack [72, 73].

Moreover, nicotinamide adenine dinucleotide (NAD^+^), while less predominantly observed in SAR, has been shown to be transported to systemic tissues to stimulate defence responses [74]. Exogenous application of NAD(P) ^+^ was shown to stimulate SAR-like responses in *Arabidopsis*, which was reinforced by the observation that the induction of SAR-like responses were inhibited in deletion mutants of *LecRK-VI.2*, a NAD(P)^+^-binding receptor [74]. Additionally, AzA has also been shown to be involved in systemic defence, via upstream SA and NHP signalling [75]. The production of glycerol-3-phosphate (G3P) is also implicated in AzA-mediated resistance and G3P does not act independently of AzA [76, 77]. As well as being induced by NHP/Pip, AzA may support SAR by acting in its positive feedback systemically [63, 75, 78].

#### Systemic aspects of systemic acquired resistance

Systemic resistance in SAR is activated upon recognition of the mobile signal [79]. While very little is known about how the mobile signal is perceived, lectins act downstream of Pip/NHP and G3P (Fig. 1a). Specifically, LEGUME LECTIN LIKE PROTEIN 1 (LLP1) is necessary for the perception of SAR [53, 80]. Once recognized, the downstream processes of SAR such as the production of SA and the NPR1-mediated activation of PR gene expression are shared with the plant’s localized response [22, 81]. The induction of NPR1 through Pip seems to provide positive feedback to both the synthesis of SA and NHP, as well as their regulators [70]. Additional inactive forms of SA stored in vacuoles may be readily hydrolysed into active SA and mobilised into other cellular locations [82].

Exclusively in distal tissues, NPR1 undergoes phosphorylation to co-activate downstream gene expression [83] and interacts with various TFs, including TGAs and WRKYs, to co-activate downstream expression [22, 69, 84, 85]. Surprisingly, the previously mentioned phosphorylation acts to mediate the degradation of NPR1 to fully activate SAR genes [86]. Klessig et al. [87] proposed that NPR1 is active in systemic tissues due to the more moderate accumulation of SA compared to localized sites. These intermediate levels of SA would be high enough to disrupt the interaction of NPR1 with NPR4 but too low to promote NPR3-associated degradation [87], leaving the cells in a primed state for rapid response to infection.

### Induced systemic resistance

Much like SAR, ISR also activates defence in systemic tissues, typically through the actions of beneficial microbes in the rhizosphere [47]. Several seminal experiments established ISR to be mediated by plant growth-promoting rhizobacteria (PGPR), typically within the *Pseudomonas* genera [88, 89]. This phenomenon is referred to as priming, where changes in gene expression are seen only after a secondary pathogen is inoculated in systemic tissues. These changes normally occur much faster than if the tissues were not induced with ISR. As reviewed by Pieterse et al. [47], several other studies revealed that various PGPR and plant growth-promoting fungi (PGPF) rely on JA/Et signalling *in planta*, thus ISR is dependent on the production of these phytohormones (Fig. 1b).

Unlike the SAR pathway, the mobile signals generated in the roots required to be translocated to systemic tissues have not yet been identified [47, 90]. In the search for mobile signals, various genes and molecules, apart from JA/Et signalling at the root interface, have been identified. MYB72 was identified as a root-specific TF necessary for the onset of ISR induced by *P. fluorescens* WCS417r and *Trichoderma* [91, 92]. Additionally, study on the metabolome of non-mycorrhizal and mycorrhizal plants during pathogen infection have indicated that improved resistance to infection at the shoots may be mediated by lignans and oxylipins (Fig. 1b), which are suggested to contribute to the mobile active signals for primed immune responses in ISR [93]. Importantly, in plants where ISR has been induced, JA/Et-dependent gene expression was induced only after subsequent challenge with a secondary pathogen [91]; instead of large-scale transcriptional reprogramming, distal tissues become more sensitive to changes in JA/Et and become primed for a faster, stronger secondary response (Fig. 1b).

Despite the disparate mechanisms of ISR and SAR, studies have revealed that their molecular mechanisms of signalling may not be so distinct [94–97]. Studies in model organisms have identified AzA, LLP2 and G3P to be involved in the induction of ISR, as depicted in Fig. 1b [94, 95, 98, 99]. A possible explanation for ISR also being active against (hemi-)biotrophic microbes is the reliance of this signalling on G3P, AZI1 and LLP2 independent of SA [9] and the downstream responses of ISR are reliant on the non-SA related function of NPR1 [100]. Well summarized by Vlot et al. [9]., various examples of ISR also implicate the role of SA and seem to not conform to the SA-JA antagonism. However, the cytosolic role of NPR1 in ISR distinctly separates the downstream signalling from SAR [101, 102].

### The interaction between the systemic acquired resistance and induced systemic resistance pathways

SAR appears to be associated with the SA defence pathway, while ISR appears to be associated with the JA/Et pathway. While SA and JA/Et tend to be antagonistic to each other [6], this may not have the same effect when it comes to systemic tissues. Little is understood about the role of SA-JA crosstalk in ISR, but synergism is thought to play a role, depending on the inducer and the host [103, 104]. Importantly, NPR1 serves as an integral part of differentiating ISR and SAR. For example, sumoylation of NPR1 activated by SA shifts the association of NPR1 from WRKY70, which acts as repressor of JA defences while promoting SA defences [105], to TGA3, a transcriptional activator [106]. The physical location of NPR1 in the cytosol or nucleus also plays a role in which pathway is activated [101].

A novel interaction between SA and JA signalling was identified by Singh and Nandi [107]. Prior to the study, OXIDATION-RELATED ZINC FINGER1 (OZF1) was known to be involved in NPR1-related and -independent SA signalling. When OZF1 was overexpressed in *Arabidopsis* infected with *Botrytis cinerea*, this study identified that there was an increased expression of JA-responsive genes. More specifically, expression of *PDF1.2, THI2.1,* and *VSP2 *was upregulated in response to this challenge. Interestingly, SA treatment was also able to trigger the expression of *AtOZF1* in *AtOZF1* mutants [107]. These findings underline the highly intricate interactions between the phytohormones involved in plant immunity.

In 2000, SAR and ISR were formally classed as synonymous [2]. However, this study and many before class systemic responses as SAR when the systemic responses are elicited by a pathogen and/or are SA-dependent, and ISR when these responses are elicited by beneficial microbes and/or are independent of SA [47]. The complexity of SAR and ISR and their crosstalk makes it difficult to fully separate these processes, thus the classification of systemic resistance often depends on the species of the host and microbial inducer [9]. The seemingly similar mechanisms underlying ISR and SAR warrant extensive further investigation to identify mechanisms that discern them.

### Systemic induced susceptibility

Not all microbial interactions eliciting systemic responses result in immunity. Processes like mechanical wounding may lead to localised resistance but systemic susceptibility [108]. Termed induced systemic susceptibility (ISS) or systemic induced susceptibility (SIS) depending on different studies, systemic immune signalling has been shown to also result in increased susceptibility in plants and its mechanisms remain elusive. SIS can be activated at various points within plant defence pathways and depends highly on the host genotype, timing of defences and the lifestyle of secondary pathogens [108].

In most cases the underlying genes targeted during susceptibility are crucial to the distinction of different defence pathways. The TF WRKY70 is targeted and increased by *Fusarium oxysporum* and results in the suppression of SA responses and susceptibility [109]. In some instances, SIS may be due to the shifting of defences from one signalling pathway to another i.e., from an SA/JA balance towards JA, which may provide resistance to other challengers, such as herbivory, at the cost of resistance to microbial pathogens [110]. Regardless of the specific changes at various levels within the plant immune system, SIS results in the change of the microbial community to induce secondary infection [111].

### Priming underlies all known forms of systemic resistance

The first evidence of defence priming being implicated in induced immunity came in 1982 [4], yet the confirmation of priming in all types of systemic immunity came much later [112]. The molecular mechanisms of priming overarch much of the localized and systemic defence response. As reviewed by Reimer-Michalski and Conrath [113], priming can result in the accumulation of PRRs, dormant signalling cascades or TFs. All result in the faster recognition of infection and activation of downstream defence responses upon future pathogen infections [113, 114].

In *Arabidopsis*, the induction of SAR by *Pseudomonas syringae* infection facilitates priming to allow for such enhanced defence responses to secondary infection. SA is critical to the priming of immune responses through its involvement in suppressing systemic JA responses, downregulation of photosynthesis in distal leaves and downregulation of growth [66]. SA, alongside Pip/NHP, also contributes to the priming of distal defence responses, including PR1 and camalexin accumulation. Pip contributes to defence priming in a SA-independent manner, through enhanced activation of ALD1 and FMO1 [66]. Importantly, FMO1 is responsible for the mediation of NHP biosynthesis; increased NHP levels in systemic tissues promotes NPR1-dependent transcriptional reprogramming to prime immune responses [67].

An important aspect of priming is that genes primed for a response will only be upregulated upon a secondary challenge with a pathogen [115]. Using multiple priming RNA-Seq datasets, researchers have been able to identify a conserved set of transcriptional changes indicative of the primed state within *Arabidopsis* [116]. These changes include the upregulation of MAPKs, TFs that enable defence (such as WRKY18), and genes related to monoterpene synthesis (like GPS1). The data meta-analysis included RNA-Seq data generated from *Vitis vinifera* treated with the beneficial microorganism *Trichoderma harzianum* T39; transcriptional reprogramming of systemic tissues to prime immunity occurred, including the enhanced expression of PRs and stilbene synthesis [117]. Additionally, RNA-Seq data of priming in leaf tissue of *A. thaliana* highlighted the importance of NHP-mediated mechanisms, as described above [66]. These findings have been reinforced by later studies, including one wherein exogenous NHP treatment induced SAR-like responses via NPR1 and facilitated a priming response in distal tissues in *Arabidopsis.* This study also identified the involvement of several TGA TFs that are critical to the NHP-associated transcriptional reprogramming that occurs during priming [118].

Priming has been associated with chromatin remodelling by histone regulation, suggesting another possible mechanism in TF signalling and downstream gene expression that is inherited [115]. Three key stages exist in this process; (i) priming (perception of a stimulus), (ii) challenged primed state (after secondary infection) and (iii) primed state through transgenerational descent [119]. In the first stage, changes in primary metabolites, such as sugars and amino acids, are most common. Following a secondary infection, in the second stage, there is a distinct reliance of the plant on SA and MAPK pathways, in addition to the accumulation of ROS and PR1. Subsequently, the transgenerational primed state is reliant on epigenetic modifications that are passed from parent to progeny [119].

## How do responses differ in tree species?

Much like their herbaceous counterparts, woody species share many aspects of the plant immune system. However, due to differences in lifestyle and properties, the mechanisms are likely to differ. The induced defence system is particularly relevant for tree species due to lower resource costs when compared to constitutive defence [120, 121]. The long lifespan of a tree, compared to the relatively shorter lifespan of their pathogens, makes the rapidly induced nature of the broad-spectrum resistance of systemic resistance an attractive mechanism to investigate [120]. Most studies on systemic resistance in trees have focused on coniferous species with a lesser focus on the angiosperm species. Figure 1 provides a simplified overview of the known and potential systemic responses a tree could induce based on various interactions.

In tree species, the understanding of the different types of systemic resistance is far less understood. While many examples of SAR and ISR under the control of SA and JA, respectively, occur in trees, trees also exhibit unique types of systemic resistance, such as systemic induced resistance (SIR). SIR, which is typically elicited by necrotrophic organisms, is viewed separately to SAR. Their distinction is because of the unknown signalling mechanisms of SIR, as depicted in Fig. 1c [120, 122, 123]. The involvement of phytohormones in SIR also remains unknown, thus the need for a distinct type of systemic response.

Although there are noticeable differences between the defence mechanisms in trees and model species, there are certainly identifiable similarities. Similarly to herbaceous plants, various PRs also play a role in tree defence [124]. While most studies have focused on the role of PRs in localized defence, far less have studied their role in systemic defence. One study identified a systemic increase in peroxidase (PR9) in response to root infection of *Picea abies* seedlings by *Ceratocystis polonica* [125]. NPR1 has also been implicated in systemic resistance. Stable antimicrobial peptide treatment of citrus trees to prevent *Candidatus Liberibacter asiaticus* infection resulted in an increased level of expression of NPR1, including in uninfected trees, suggestive of a primed systemic response [126].

## Systemic resistance in trees

In herbaceous plants, systemic signalling mechanisms have mostly been studied through the use of mutants, grafting and transgenic approaches [58, 127, 128]. While great strides have been made in the genetic engineering of trees to study their signalling mechanisms, relatively low transformation efficiencies and difficulties in regeneration means that its use in research is still limited [129]. Most studies have relied on other methods to determine endogenous signals in systemic responses. Most commonly, the application of exogenous inducers of systemic defence i.e., SA and MeJA, followed by observing tree responses, is used. Alternatively, sub-lethal inoculation is used to elicit a response for analysis. Most of these studies have focused on the inducers’ effect at the localized tissue, which often exhibit unique defences not shared with their herbaceous counterparts as previously described. Other studies have identified signalling molecules induced in systemic tissues which give insight into how trees may share systemic signalling molecules with herbaceous plants.

### Examples of systemic resistance in conifer species

SIR was first described in a variety of *Pinus spp.*; in-field observations of *P. radiata* showed a SIR phenotype of lesions caused by *Fusarium circinatum* decreasing over time [122, 130]. Another classic example of SIR in conifers is the interaction of *P. nigra* with *Diplodia pinea*, which is characterized by increases in phenolics, stilbenes and lignin deposition, shown in Fig. 1c [131, 132]. Later studies confirmed the association of increases in lignin and other phenolic compounds with SIR [133]. Importantly, this pattern is not observed equally across tree tissues. The concentration of monoterpenes and diterpenes, among others, have been shown to remain at very low levels in constitutive and systemic phloem, whereas at the localised point of infection, levels increased by up to 300-fold [134, 135]. Elicitation of stronger localised than systemic responses suggests that the cost of priming for later attack is too great.

While pine species tend to respond to pests and pest-associated fungi in a very localized manner, their response to necrotrophs differ. When inoculated with *Sphaeropsis sapinea* and *Diplodia scrobiculata*, *P. nigra* has been shown to induce lignification and the production of several phenolic compounds and secondary metabolites in systemic tissues, which ultimately reduced disease severity upon a re-infection with the same pathogen, respectively [132]. Over time, these observations have been substantiated through phytohormone profiling and more extensive metabolite and phytochemical analysis [136, 137].

It is notable that SIR and SIS have been shown to occur concurrently in *P. nigra* in a bi-directional and organ-dependent manner, whereby induction at the stem base resulted in SIS at shoot tips, while induction at the lower stem resulted in SIR in the upper stem [132]. This could be explained by the energy costs of induced systemic resistance during infection; should the induction event severely damage the host’s defences, energy reserves would be depleted and result in SIS. This is known as the SIR hypothesis. Sherwood and Bonello [138] aimed to identify where and when SIS would occur instead of SIR in *P. nigra*, as defined by the SIR hypothesis. SIR appeared to increase in strength over time in stems, while only SIS was observed in the shoots. This unique phenomenon has not been studied in other tree or plant species but is an interesting avenue for further study.

In addition to the seminal work on SIR, various *Pinus spp.* have exhibited reliance on more traditional pathways of defence as well [139, 140]. Possible SAR and ISR have been observed in conifers, including in *P. radiata*, where *Clonostachys rosea* was shown to induce ISR against the fungal pathogen *F. circinatum* [141] and in *P. albicaulis*, where pine transcriptomes of MeJA- and *Cronartium ribicola-*induced systemic resistance were compared [142]. The type of resistance elicited by *C. ribicola* overlapped significantly with the MeJA-induced ISR. Additionally, many of the significantly differentially expressed genes were found to correlate well with other *Pinus* genes, suggesting lineage-specific gene expression. These genes mainly belonged to the *PR* genes and secondary metabolism such as phenolics and terpenes [142]. These findings are reinforced by a later study where *P. sylvestris* treated with JA identified increases in total phenol content and carotenoid content [143].

Similarly, the systemic responses elicited by MeJA in various *Picea spp.* have also been investigated. In Norway spruce (*P. abies*), the induction of systemic defences through fungal infection or MeJA treatment was able to protect the trees against insect damage [144]. In addition to changes in terpene levels, the levels of chitinase (Chi4) and peroxidase (PX3) were significantly upregulated upon biotic challenge with the fungus *Endoconidiophora polonica* or MeJA, compared to the control (Fig. 1c). Fungal infection resulted in a rapid increase of defence genes that was prolonged to provide protection upon secondary exposure to insects. In contrast, MeJA-treated trees showed a minimal increase in these genes prior to insect exposure and showed rapid increases to far higher rates after exposure. Surprisingly, while the induction of defences led to more insect entry holes, infestation severity was limited [144].

Researchers have probed further into understanding the mechanisms of the MeJA priming phenomenon in *P. abies* [145]. By using MeJA as a pre-treatment followed by wounding to elicit defence responses, the effect of MeJA priming on subsequent resistance could be observed. Upon the stimulus of wounding, various terpenes and JA, but not SA, were significantly upregulated. Interestingly, following MeJA treatment, evidence of epigenetic modulation was observed as a possible link to the preparation phase of priming. Unlike terpene genes previously identified as important in this interaction, the priming of PR genes, such as chitinase, under MeJA treatment was further validated [145].

It is important to note that plant responses to the application of phytohormones does not always match the responses elicited when biologically induced. This was illustrated in *P. contorta* populations that had historically been exposed to *Grosmannia clavigera* could distinguish between the pathogen and artificially applied JA, as reflected in the minimal induction of defensive monoterpenes in MeJA-treated plants [146]. Contrastingly, populations that had not been exposed to the pathogen could not distinguish between *G. clavigera* infection and external JA application, likely a result of co-evolution of the plant and pathogen over time.

Furthermore, the induced response of the tree to a secondary biotic stress is not only determined by the inciting agent, but the manner in which SIR is incited as well; plant responses have been shown to vary, dependent on whether they are mechanically-, herbivore- or infection-induced [147]. For example, prior infection of *Larix decidua* with *Mycosphaerella laricinia* resulted in SIR, demonstrated by reduced Larch sawfly feeding one year later, whereas prior herbivore- and mechanically-induced defoliation did not [147].

### Examples of systemic responses in angiosperm species

Far less research has been conducted on systemic responses in angiosperm trees, but it is logical to presume that a similar evolution to the conifers may have occurred. It is, however, expected that the trees have distinctive qualities that make the response of angiosperm species disparate from conifers. While conifer species are economically important, as are angiosperms and thus they remain valuable to study. From the very limited studies available we hope to elucidate the gaps in the understanding of tree-specific systemic defence in angiosperms.

In *Eucalyptus*, the role of SA is critical to the resistance and susceptibility to infection by *Chrysoporthe austroafricana*. It has been found that the external application of SA on a highly susceptible hybrid of *E. grandis* is able to induce resistance similar to the level of a moderately resistant hybrid of the same species [148]. Further validating the role of SA, Mangwanda et al. [149] showed that basal levels of SA in resistant trees were inherently higher. Upon *C. austroafricana* infection, transcriptome profiling over a time course of infection revealed differences between the clones with a possible delay in the response of the susceptible clone. Investigation of the differentially expressed genes revealed support for the up-regulation of SA signalling and SAR, but these were restricted to the resistant clone [149].

SAR has also been observed in Poplar [152]; the interaction of *Paulownia tomentosa* with *Botryosphaeria dothidea* results in the systemic accumulation of SA and MeSA in both localized and systemic tissues. Active manipulation of SA levels through MeSA breakdown in systemic tissues is also observed. The downstream expression of *PR-1*, *PR-2*, *PR-5* and *PR-10*, classic markers of SA-related downstream responses, are also up-regulated [152]. The role of SA in *P. tomentosa*-*B. dothidea* was further validated as a prominent phenolic compound when compared to a susceptible species [153]. Moreover, functional genetics has begun to reveal the role of some components of SAR in Poplar, such as the role of salicylate methyltransferase in the production of MeSA from SA [154].

Evidently SAR is a vital component of systemic defence in angiosperms, as argument has been strengthened by proteomics studies [155]. However, evidence for the induction of JA/Et signaling and other resistive mechanisms suggests that it is not a solely SAR-like response that is induced by *E. grandis* and that it may employ various systemic responses, including ISR and SIR. For example, ISR induced by PGPR has also been observed in *Eucalyptus* species. *E. grandis* treated with *Streptomyces* led to improved resistance to later infection by *Botrytis cinerea* [151]. Similarly, PGPRs, specifically *Bacillus subtilis*, have also been reported to promote ISR in apple trees, to prevent *Fusarium spp.* infection [156]. These responses have been further confirmed in other apple rootstocks planted in soil with arbuscular mycorrhizal fungi; several defence-related enzymes, including superoxide dismutase and other antioxidants, were upregulated in distal tissues [157].

The induced defence elicited by *Streptomyces sp.* PM9 strain pre-treatment was evaluated in *E. grandis* and *E. globulus* [151]. Subsequently, pre-treated plants were also inoculated with *B. cinerea.* The symptoms of *B. cinerea* were delayed in *E. grandis* but not *E. globulus*. This was attributed to the relatively earlier upregulation of peroxidase activity prior to symptom development as well as the synthesis of phenolics such as flavonoids and 2-hydroxybenzoic acid, a structural analogue of SA [151]. In oak, pre-treatment of the roots with the bacterial strain AcH 505 reduced the infection of oak powdery mildew on leaves of infected trees [150]. Not only did AcH 505 prime oak for a heightened response, the responses seemed to involve components of both ISR and SAR, along with regulation by ABA [150].

### Systemic induced susceptibility in trees

While most studies of systemic responses in trees investigate resistance, it is important to note that SIS may still occur. Organ-dependent development of systemic defences in *P. nigra* revealed that while SIR is expressed in some systemic tissues, others develop SIS [132]. Sherwood and Bonello [138] further postulated that all examples of SIR can result in SIS over time if enough damage is inflicted on the host. This study modelled the interaction of *P. nigra with D. sapinea* and validated their hypothesis [138]. While the point at which systemic resistance can become systemic susceptibility can be modelled, no information regarding the molecular mechanisms of this phenomenon have been elucidated. It is important to remember the complex nature of systemic responses and their underlying signalling pathways. The development of any systemic response depends on: (1) the inducer of defences which may be living micro-organisms or chemicals, (2) the nature of the resulting systemic responses, and (3) the lifestyle of the additional pathogen challenge.

## Conclusion

Systemic responses provide broad-spectrum resistance to a variety of pests and pathogens in trees. Most critically, the core genes and phytohormones that are uniquely deployed by trees to sustain a systemic resistance to common pests and pathogens remain unclear. These responses can be particularly useful in the management of disease in trees, as they can be managed and manipulated to generate more resistant and robust trees in forestry. The use of SA and JA analogues, among others, has effectively stimulated systemic resistance in plants, preparing them to respond to biotic threat [158, 159]. However, implementing these control strategies is challenging due to the potential for their off-target effects in-field, thereby posing an environmental risk. In addition, frequent chemical treatment is restricted by regulatory bodies, such as the Forest Stewardship Council. Another concern is that the efficacy and practicality of applying these strategies on a larger scale have not been confirmed. Nonetheless, these forest management approaches hold potential for long-term benefits. Trees have long lifespans, making it more likely that they will face multiple threats during their lifetime. Therefore, properly enhancing their defence mechanisms through priming can contribute to long-term protection.

## Data Availability

Not applicable.

## Abbreviations

ABA: Abscisic acid
AzA: Azelaic acid
CKs: Cytokinins
Et: Ethylene
ETI: Effector-triggered immunity
G3P: Glycerol-3-phosphate
HR: Hypersensitive response
IR: Induced response
ISR: Induced systemic resistance
JA: Jasmonic acid
MAPKs: Mitogen-activated protein kinases
MeJA: Methyl-jasmonate
MeSA: Methyl-salicylate
NOs: Nitric oxides
NHP: N-hydroxypipecolic acid
PGPF: Plant growth-promoting fungi
PGPR: Plant-growth-promoting rhizobacteria
Pip: Pipecolic acid
PRs: Pathogenesis-related proteins
PTI: Pattern-triggered immunity
ROS: Reactive oxygen species
SA: Salicylic acid
SAR: Systemic acquired resistance
SIR: Systemic induced resistance
SIS: Systemic induced susceptibility
TF: Transcription factor
